# Assessment of the health utility of patients with leukemia in China

**DOI:** 10.1186/s12955-021-01711-1

**Published:** 2021-02-27

**Authors:** Xueyun Zeng, Mingjie Sui, Rui Liu, Xinyu Qian, Wenfeng Li, Erwei Zheng, Jinjin Yang, Jinmei Li, Weidong Huang, Hongbin Yang, Hongjuan Yu, Nan Luo

**Affiliations:** 1 School of Health Management, Harbin Medical University, Harbin, 150086 China; 2 National University Singapore Saw Swee Hock School of Public Health, Singapore, 117549 Singapore; 3 Department of Hematology, The First Affiliated Hospital of Harbin Medical University, Harbin, 150001 China; 4 First Affiliated Hospital of Harbin Medical University, Harbin, 150001 China; 5 Public Health Education and Information Center of Heilongjiang Province, Harbin, 150063 China; 6 Department of Medical Oncology, Third Affiliated Hospital of Harbin Medical University, Harbin, 150081 China; 7 Southern University of Science and Technology Hospital, Shenzhen, 518055 China

**Keywords:** Leukemia, Health utility, EQ-5D-5L, Cancer

## Abstract

**Objectives:**

This study aimed to assess the health utility of leukemia patients in China using the EQ-5D-5L, compare it with the population norms, and identify the potential factors associated with health utility.

**Methods:**

A hospital based cross-sectional survey was conducted in three tertiary hospitals from July 2015 to February 2016. A total of 186 patients with leukemia completed the EQ-5D-5L and their health utility scores were calculated using the Chinese value set. EQ-5D-5L utility and dimensions scores of leukemia patients were compared with China’s population norms using Kruskal–Wallis test and chi square test. Potential factors associated with health utility were identified using Tobit regression.

**Results:**

The mean EQ-5D-5L utility scores of patients with leukemia, grouped by either gender or age, were significantly lower than those of the general population (*p* < 0.001). The same results were found for individual dimensions of EQ-5D-5L, where leukemia patients reported more health problems than the general population (*p* < 0.001). The utility score of leukemia patients was found to be significantly related to medical insurance, religious belief, comorbidities, social support and ECOG performance status.

**Conclusion:**

This study indicated that leukemia patients have worse health status compared to the general population of China and that multiple factors affect the health utility of the patients. The utility scores reported in this study could be useful in future cost-utility analysis.

## Introduction

Leukemia is a malignant tumor that is common in both children and adults. Leukemia was estimated to be responsible for 309,006 deaths in 2018 and 437,033 new leukemia cases were diagnosed in the same year [1]. China has one of the largest leukemia populations in the world with 75,300 new cases in 2015 [2]. With novice treatment options available in China, the five-year survival rate of leukemia patients improved from 19.6% in 2003–2005 to 25.4% in 2012–2015 [3]. While survivability is favorable, surviving patients may, however, be affected by treatment toxicity, increased risk of second malignancy and side effects [4, 5]. Therefore, Health–related quality of life (HRQoL) is an important concern in patients with leukemia [6].

Disease-specific instruments like the European Organization for Research and Treatment of Cancer Quality of Life Core Questionnaire 30 (EORTC QLQ-C30), Medical Research Council/EORTC Quality of Life Questionnaire Leukemia Module (MRC/EORTC QLQ-LEU), Functional Assessment of Cancer Therapy-General (FACT-G) and the Functional Assessment of Cancer Therapy-Leukemia(FACT-Leu) are commonly used to measure the HRQoL of patients with leukemia [6–10]. While they are valid and reliable, they cannot be used to generate health utility value for leukemia patients because they are not preference-based instruments [11].

Health utility, representing the strength of an individual’s preferences for different health states, can be used to summarize HRQoL into a numeric index ranging from 0 to 1 [12]. EQ-5D is currently one of the most widely used standardized multi-attribute utility instruments (MAUIs) [13] for cost-utility analysis (CUA) of healthcare [14]. It is recommended by a number of bodies and guidelines including the National Institute for Health and Care Excellence (NICE) in the UK [15].

Three versions of EQ-5D are available including EQ-5D-Y for children, EQ-5D-3L and EQ-5D-5L for adults. EQ-5D-3L has been widely used to measure, compare and value health status across disease areas including leukemia populations [16]. EQ-5D-5L was developed to improve the measurement properties of EQ-5D-3L such as ceiling effect and sensitivity [17]. Ever since, scoring algorithms of EQ-5D-5L based on the general public’s health preferences were developed in many countries such as China [18], the UK [19] and Germany [20]. While EQ-5D-5L has been applied to cancer patients [11], It is rarely used to measure health status and health utility of patients with leukemia.

In order to fill these gaps, we therefore assessed the utility scores of leukemia patients using EQ-5D-5L and compared them with the norms for the general adult Chinese population, and identified the potential factors associated with health utility in leukemia.

### Methods

#### Study design and data collection

We consecutively recruited patients from three tertiary hospitals in Harbin, Heilongjiang province, northeast China. The hospitals are the centers for leukemia treatment in the region and therefore their leukemia patients are representative. The inclusion criteria of patients were: (1) a diagnosis of leukemia; (2) aged 18 or above; (3) able to understand the questionnaire well. After obtaining informed consent, a face-to-face interview was conducted in a private room by a trained student from Harbin Medical University. The interviews were conducted from July 2015 to February 2016. In order to ease the burdens of respondents and ensure accuracy of information, some clinical information was collected from doctors/nurses (e.g., types of leukemia). Ethical permission (*HMUIRB2014012)* was granted by the Regional Ethical Committee, Harbin Medical University.

Following suggestions in the literature [5, 7, 21–25], we developed a questionnaire with a three-part structure for information collection: (1) socio-demographic information; (2) clinical information; and (3) health status of patients. Socio-demographic information included gender, age, ethnicity, religious belief, level of education, marital status, medical insurance and annual household income. For assessing religious belief, we set a question ‘Do you have any religious belief ? (yes/no)’. Clinical information included type of leukemia, duration since diagnosis, performance status and comorbidities. The interviewer administered standardized instruments to measure the health status of patients including emotional distress with the Hospital Anxiety and Depression Scale (HADS), perceived social support with the Social Support Self-Rating Scale (SSRS), family function with the APGAR scale, and health utility with EQ-5D-5L.

#### Measurements

##### EQ-5D-5L

As a preference-based instrument, EQ-5D-5L consists of two parts: the EQ-5D-5L descriptive system and the EQ Visual Analogue Scale (EQ-VAS). With the descriptive system, EQ-5D-5L can define 3,125 (= 5^5^) health states in the context of five levels (no problems, slight problems, moderate problems, severe problems, and extreme problems) and 5 dimensions (mobility, selfcare, usual activities, pain/discomfort, anxiety/depression). Each heath state can be converted into a utility score using a country-specific value set based on social preferences [26]. In this study, we calculated health utility score of patients with leukemia using the Chinese value set which generates the maximal preference weight of 1 and the minimal preference weight of − 0.391 [18].

##### HADS

The Hospital Anxiety and Depression Scale [27] (HADS) is a self-rating scale to assess emotional distress in non-psychiatric patients. HADS measures the anxiety and depression using 14 items including 7 item for anxiety and 7 items for depression. The two subscales have the same score range from 0 to 21, with the severity of depression or anxiety being categorized as normal (0–7), mild (8–10), moderate (11–14), or severe (15–21) [28]. We used the validated Chinese version of HADS.

##### SSRS

The Social Support Self-Rating Scale (SSRS) developed by Xiao [29] is one of most frequently used instruments for measuring social support in China. SSRS consists of 10 items which form three subscales: subjective support (4 items), objective support (3 items), and utilization of social support (3 items). The total support score ranges from 12–66; the level of social support can be classified into three categories: low (≤ 22), moderate (23–44), and high (45–66).

##### APGAR

The family function of patients was measured using the validated family APGAR scale[30]. The APGAR scale consists of five questions measuring five components of family function (adaptation, partnership, growth, affection, and resolve), with three possible answers (“almost never” = 0, “sometimes” = 1, “almost always” = 2). The sum of scores range from zero to ten and family function can be classified as either severely dysfunctional (0–3), moderately dysfunctional (4–6), or highly functional (7–10).

##### ECOG

The performance status is an important part of leukemia and was accessed using the Eastern Cooperative Oncology Group (ECOG) scale. Interviewers collected records from doctors who rated the grade of patient with the range from 0 (fully functional and asymptomatic) to 4 (bed ridden) [31].

#### Statistical analysis

We described the basic characteristics of the sample using mean and standard deviation (SD) for continuous variables and frequency and percentage for categorical variables. The distribution of the EQ-5D-5L health utility score was skewed, therefore, the Wilcoxon signed-rank test was used to compare the utility score of respondents with the general Chinese population [32]. Since the age-gender profiles of the patients and the general population were different, we used the normative data to calculate expected mean utility scores for the patients by adjusting for age and gender [33]. In addition, patients’ responses to the five dimensions of EQ-5D-5L were compared to the general population norms and the significance of the difference was determined using the Chi-square test.

To explore the factors associated with utility score, we compared the utility scores of patients with different socio-demographics (gender, age, ethnicity, religious belief, level of education, marital status, medical insurance, annual household income), clinical characteristics (types of leukemia, duration since diagnosis, ECOG score, comorbidities), and psychosocial characteristics (anxiety, depression, social support, family function). The variables that were significantly associated with the health utility score in the Kruskal–Wallis test (*p* < 0.05) will be entered to a Tobit regression model. Despite the reduced ceiling effect of using EQ-5D-5L, 23.5% of the patients reported full health (11,111). We therefore selected the Tobit regression model to address the distribution of censored data [34].

### Results

#### Basics of sample

Of 208 eligible patients with acute myeloid leukemia (AML) and acute lymphoblastic leukemia (ALL), 22 patients refused to complete the survey with the following reasons: 10 patients felt ‘uncomfortable’, 8 patients ‘lacked interest’, and 4 patients did not ‘understand the informed consent’. In the end, 186 patients with leukemia completed the questionnaires independently.

The mean age of patients was 46 years old and the average duration since diagnosis was 21.7 months. The majority was Han nationality (96.2%), married (81.2%), had religious belief (88.2%) and medical insurance (91.4%). Most of them had middle or high school qualifications (59.7%), and reported annual household income ranging from 40,001 to 79,999 Chinese Yuan (approximately 5700 to 11,400 in US Dollar) (48.9%). The mean score of the patients was 10.9 for anxiety, 7.9 for depression, 37.2 for social support, and 6.9 for family function (Table 2).

#### Utility scores of leukemia patients versus population norms

**Fig. 1:**
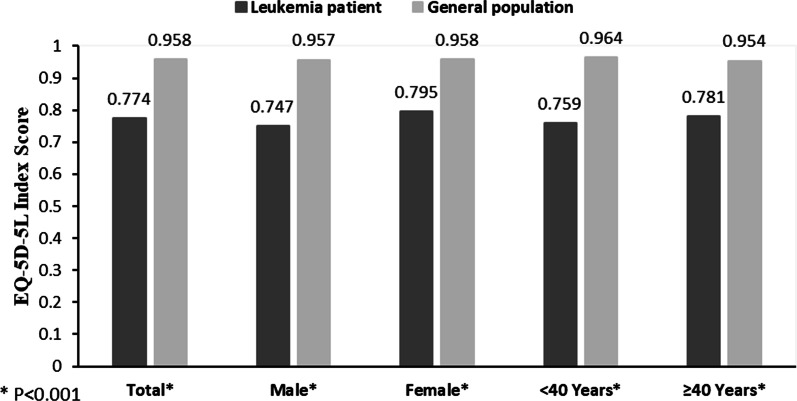
Utility scores of leukemia patients compared with population norms

Figure 1 depicts the comparison of utility scores between leukemia patients and population norms for EQ-5D-5L in China. Patients had a significantly lower utility score than the population norms (0.774 vs. 0.958, *p* < 0.001). The utility score of patients with leukemia grouped by gender and age were also significantly lower than and- and gender-specific population norms (*p* < 0.001).

#### EQ‑5D‑5L dimensions

The highest proportion of the ‘no problems’ response was observed in the ‘self-care’ dimension (62.9%), followed by ‘usual activities’ (62.4%), ‘mobility’ (62.4%), ‘pain/discomfort’ (47.8%), and ‘anxiety/depression’ (39.8%). A total of 45 patients (24.2%) reported no problems in all five dimensions. The proportion of leukemia patients who reported problems in the five dimensions was significantly higher than that of the general population (Table 1).

**Table 1:** Frequency of item response in each EQ-5D-5L dimension compared with the general

Problems	Mobility (%)*		Self-care (%)*		Usual activities (%)*		Pain/Discomfort (%)*		Anxiety/Depression (%)*	
	Leukemia patients	General population	Leukemia patients	General population	Leukemia patients	General population	Leukemia patients	General population	Leukemia patients	General population
No problems	62.4	94.7	62.9	98.8	62.4	96.0	47.8	68.9	39.8	73.5
Slight problems	31.7	4.5	31.2	0.9	23.1	3.7	26.3	28.8	28.0	23.7
Moderate problems	3.8	0.6	3.2	0.2	8.1	0.2	16.1	1.7	16.1	2.5
Severe problems	1.1	0.2	2.2	0.2	4.3	0.2	4.8	0.5	8.6	0.2
Extreme problems	1.1	0	0.5	0	2.2	0	4.8	0.2	7.5	0.2

*p < 0.001 based on the Chi-square test

#### Univariate analysis of utility score

The overall EQ-5D-5L utility mean score was 0.774, while the mean EQ-VAS score was 74.4. The utility scores were higher among those with religious belief  (*p* = 0.036), had medical insurance (*p* = 0.001), and did not had comorbidities (*p* < 0.001). In addition, those patients with lower social support (*p* < 0.001), higher family function score (*p* < 0.001), and higher ECOG performance status (*p* < 0.001) had lower utility scores (Table 2).

**Table 2:** Utility scores of EQ-5D-5L of respondents in different characteristics

	N = 186	Mean ± SD	Median(range)	95% CI	*P* values
*Gender*					0.248
Male	86 (46.2%)	0.751 ± 0.274	0.846 (− 0.222–1)	0.692–0.809	
Female	100 (53.8%)	0.795 ± 0.246	0.882 (− 0.251–1)	0.746–0.844	
*Age (years, mean ± SD)*	45.77 ± 14.30				0.584
≤ 37	60 (32.3%)	0.759 ± 0.289	0.848 (− 0.251–1)	0.685–0.834	
> 37	126 (67.7%)	0.782 ± 0.245	0.882 (− 0.222–1)	0.738–0.825	
*Ethnicity*					0.453
Han	179 (96.2%)	0.777 ± 0.260	0.882 (− 0.251–1)	0.739–0.815	
Other	7 (3.8%)	0.702 ± 0.266	0.771 (0.329–1)	0.456–0.948	
*Religious belief*					0.036*
No	164 (88.2%)	0.764 ± 0.267	0.848 (− 0.251–1)	0.723–0.805	
Yes	22 (11.8%)	0.849 ± 0.187	0.893 (0.262–1)	0.767–0.932	
*Level of education*					0.916
No more than primary school	31(16.7%)	0.757 ± 0.237	0.848 (-0.017–1)	0.670–0.843	
Middle or high school	111 (59.7%)	0.779 ± 0.280	0.893 (− 0.251–1)	0.726–0.831	
University	44 (23.7%)	0.776 ± 0.222	0.804 (0.083–1)	0.709–0.844	
*Marital status*					0.731
Married	151 (81.2%)	0.778 ± 0.256	0.882 (− 0.251–1)	0.736–0.819	
Other	35 (18.8%)	0.761 ± 0.277	0.848 (0.022–1)	0.668–0.856	
*Medical insurance*					< 0.001**
No	16 (8.6%)	0.400 ± 0.454	0.345 (− 0.251–1)	0.158–0.642	
Yes	170 (91.4%)	0.810 ± 0.202	0.882 (− 0.164–1)	0.779–0.840	
*Annual household income(CNY¥/USD $)*					0.223
≤ 40,000 /5700	86 (46.2%)	0.741 ± 0.284	0.848 (− 0.251–1)	0.681–0.802	
40,001/5700–79,999/11,400	91 (48.9%)	0.797 ± 0.242	0.893 (− 0.222–1)	0.747–0.848	
≥ 80,000/11,000	9 (4.8%)	0.857 ± 0.132	0.894 (0.634–1)	0.755–0.959	
*Types of leukemia*					0.066
ALL	18 (9.7%)	0.668 ± 0.3	0.748 (− 0.222–1)	0.519–0.817	
AML	168 (90.3%)	0.786 ± 0.253	0.889 (− 0.251–1)	0.747–0.824	
*Duration since diagnosis*	21.67 ± 20.16				0.857
≤ 12	84 (45.2%)	0.774 ± 0.275	0.893 (− 0.251–1)	0.715–0.834	
13–24	45 (24.2%)	0.758 ± 0.280	0.882 (− 0.222–1)	0.674–0.842	
≥ 24	57 (30.6%)	0.787 ± 0.220	0.831 (0.022–1)	0.729–0.845	
Anxiety	10.97 ± 2.29				0.236
Normal	11 (5.9%)	0.923 ± 0.087	0.951 3333333433 (0.738–1)	0.865–0.982	
Mild	72 (38.7%)	0.776 ± 0.307	0.902 (− 0.251–1)	0.704–0.849	
Moderate	90 (48.4%)	0.761 ± 0.225	0.804 (− 0.160–1)	0.713–0.808	
Severe	13 (7%)	0.731 ± 0.272	0.699 (0.312–1)	0.567–0.896	
Depression	7.9 ± 2.21				0.979
Normal	83 (44.6%)	0.779 ± 0.262	0.841 (− 0.251–1)	0.721–0.836	
Mild	81 (43.5%)	0.770 ± 0.262	0.885 (− 0.222–1)	0.712–0.828	
Moderate	22 (11.8%)	0.773 ± 0.253	0.871 (0.083–1)	0.662–0.886	
Social support	37.21 ± 7.97				< 0.001**
Low	8 (4.3%)	0.623 ± 0.108	0.632 (0.493–1)	0.533–0.713	
Moderate	133 (71.5%)	0.735 ± 0.276	0.824 (− 0.251–1)	0.688–0.783	
High	45 (24.2%)	0.917 ± 0.155	1.0 (0.262–1)	0.870–0.963	
Family function (APGAR score) (Mean ± SD)	6.86 ± 1.81				< 0.001**
*Severely dysfunctional (n, %)*	47 (25.3%)	0.698 ± 0.246	0.734 (− 0.164–1)	0.848–0.937	
Moderate dysfunctional (n, %)	89 (47.8%)	0.749 ± 0.291	0.848 (− 0.251–1)	0.687–0.810	
Highly functional (n, %)	50 (26.9%)	0.892 ± 0.156	0.947 (0.262–1)	0.625–0.770	
*ECOG score*					< 0.001**
0	43 (23.1%)	0.939 ± 0.133	1.0 (0.475–1)	0.898–0.979	
1	97 (52.2%)	0.794 ± 0.203	0.893 (0.083–1)	0.753–0.835	
2	34 (18.3%)	0.643 ± 0.237	0.725 (− 0.017–1)	0.560–0.725	
3	12 (6.5%)	0.403 ± 0.475	0.520 (− 0.251–1)	0.102–0.705	
*Comorbidities*					< 0.001**
No	136 (73.1%)	0.836 ± 0.184	0.893 (0.022–1)	0.805–0.868	
Yes	50 (26.9%)	0.606 ± 0.348	0.585 (− 0.251–1)	0.506–0.705	

^*^p < 0.05 and **p < 0.01, respectively

CNY¥ represents Chinese Yuan; USD $ represents US dollar

#### Factors associated with health utility score

The results of Tobit regression analysis are displayed in Table 3. Higher utility score was significantly associated with religious belief (*p* = 0.017), medical insurance (*p* = 0.003), absence of comorbidities (*p* = 0.002), higher level of social support (*p* = 0.007), and lower ECOG performance status (*p* = 0.016).

**Table 3:** Influencing factors of EQ-5D-5L utility scores from Tobit regression model

Variables	Coefficient	SE	*P* values
*Religious belief*			
Yes	Ref		
No	− 0.075	0.031	0.017*
*Medical insurance*			
Yes	Ref		
No	− 0.293	0.097	0.003*
*Comorbidities*			
No	Ref		
Yes	− 0.133	0.038	0.001*
*Type of leukemia*			
AML	Ref		
ALL	− 0.102	0.065	0.119
*Social support*			
High	Ref		
Moderate	− 0.117	0.043	0.007*
Low	− 0.309	0.115	0.008*
*Family function (APGAR score)*			
Highly functional	Ref		
Moderate dysfunctional	− 0.041	0.039	0.296
Severely dysfunctional	− 0.106	0.055	0.056
*ECOG score*			
0	Ref		
1	− 0.302	0.124	0.016*
2	− 0.396	0.153	0.010*
3	− 0.514	0.194	0.009*

### Discussion

To the best of our knowledge, this study was the first to examine the utility score of patients with leukemia using EQ-5D-5L in China. Being a malignant tumor, leukemia-related policy development is increasingly opting for CUA to assist health resource allocation. We calculated the utility scores of leukemia patients using a representative sample of leukemia with a Chinese value set. Therefore, the baseline utility score could be used for calculating the quality-adjusted life year (QALY) which is a central input in the CUA of health care for leukemia.

The mean utility of patients, even after adjustment of age or gender, was lower than the general Chinese population. This is similar to the finding in a prior study of patients with CLL in Netherlands population [22]. Other studies of leukemia patients using FACT-G [7] and EQ-VAS [35] shown similar trend. The mean utility score (0.778) in this study was a little lower than the mean of utility (0.81) [22] in patients from Netherland. The possible reasons for this difference could be due to cultural, clinical and socioeconomic characteristics of the two samples. Furthermore, utility scores calculated in this study could be more accurate that scores derived from mapping in previous studies [22]. In addition, the patients reported more problems in all five dimensions than general population. The current results together with prior related studies highlighted the negative impact of leukemia on HRQoL of patients.

Religious belief could be an important aspect that help cancer patients find meaning in life [36], get support [37], and provide comfort [38]. A recent meta-analysis found that greater religious belief is associated with better HRQoL in cancer patients including those with leukemia [39]. This relationship was also confirmed in the current study. These results underscore the importance of patients’ religion as part of comprehensive leukemia care. China is a multi-religion country and the number of religious believers is growing exponentially, despite being relatively lower compared to other Asian countries [40]. Given that the finding about the relationship between religion and HRQoL in the leukemia population is limited in China, further empirical research is needed.

The finding that patients not covered by medical insurance reported lower health utility was consistent with previous studies in leukemia [41] and other solid tumors [34]. Although prognosis of leukemia is relatively less detrimental than other forms of cancer, it requires long-term treatment, rehabilitation, and care which could be costly [41, 42]. The protecting effect of medical insurance could be due to the peaceful mind it brings to the patients. Patients with medical insurance might have experienced extra mental stress and fear which negatively affected their HRQoL [43, 44]. Moreover, it is possible that patients with medical insurance received novel treatments that are costly but more effective. Unfortunately, we are not able to test this hypothesis because of unavailability of treatment data. Future studies should further investigate the effect of medical insurance on patients’ HRQoL.

In the treatment of leukemia, patients often were accompanied by comorbidities such as infection, bleeding, anemia, and fatigue. These comorbidities could deteriorate the health of patients and their HRQoL. In addition, the results also confirmed that HRQoL worsens with lower ECOG performance status. These findings were in line with prior limited data exist on the relationships between comorbidity, ECOG performance, and HRQoL for leukemia patients [45]. Therefore, these results in the current study indicated that the ECOG and comorbidities can be robust predictors of HRQoL and offer information to assist clinicians in decision-making.

In some previous studies, social support had been identified as an important factor associated with improved quality of life for some solid cancer patients (e.g., breast cancer [46] and lung cancer [47]). Compared to the cancer population with solid tumor, individuals with leukemia are at higher risk of potential life-threatening experience, prolonged treatment with adverse effects, and catastrophic economic burden [23, 25]. In line with a few studies in which leukemia patients with higher levels of social support were found to have better HRQoL [48, 49], the present study found perceived social support to be an important predictor of better HRQoL. Therefore, it is essential to build a good social support network for the vulnerable leukemia population.

### limitations

There were some limitations in this study. First, this study surveyed inpatients with leukemia in tertiary hospitals without inclusion of outpatients. Second, our study did not include patients with CML and CLL which are rare types of leukemia in Chinese adults and our study sample is at best only regionally representative. Therefore, the findings of our study may not be generalizable to other geographic areas of the country. Third, what we observed are associations between characteristics and HRQoL of leukemia patients in a context of cross-sectional survey. Causal relationships should be investigated in future longitudinal studies. Lastly, some potential factors of HRQoL such as cancer stage and therapeutic modalities were not investigated. Those should be explored in future studies.

### Conclusion

This study indicated that leukemia patients have lower health utility and poor health status compared to the general Chinese population and that there are multiple factors affecting the patients’ health utility. Given the limited health utility data for leukemia patients, the utility scores reported in this study could be useful in future cost-utility analysis (CUA) of treatments for leukemia.

## Data Availability

Data can be obtained from the corresponding author under reasonable request.
